# On-Chip Real-Time Chemical Sensors Based on Water-Immersion-Objective Pumped Whispering-Gallery-Mode Microdisk Laser

**DOI:** 10.3390/nano9030479

**Published:** 2019-03-24

**Authors:** Qijing Lu, Xiaogang Chen, Liang Fu, Shusen Xie, Xiang Wu

**Affiliations:** 1 Key Laboratory of Optoelectronic Science and Technology for Medicine of Ministry of Education, Provincial Key Laboratory for Photonics Technology, Institute of Laser and Optoelectronics Technology, Fujian Normal University, Fuzhou 350007, China; qjlu@fjnu.edu.cn (Q.L.); xgchen01@139.com (X.C.); fuliang1995@126.com (L.F.); 2 Department of Optical Science and Engineering, Fudan University, Shanghai 200433, China

**Keywords:** whispering-gallery-mode (WGM), sensor, water-immersion-objective (WIO), microdisk, laser

## Abstract

Optical whispering-gallery-mode (WGM) microresonator-based sensors with high sensitivity and low detection limit down to single unlabeled biomolecules show high potential for disease diagnosis and clinical application. However, most WGM microresonator-based sensors, which are packed in a microfluidic cell, are a “closed” sensing configuration that prevents changing and sensing the surrounding liquid refractive index (RI) of the microresonator immediately. Here, we present an “open” sensing configuration in which the WGM microdisk laser is directly covered by a water droplet and pumped by a water-immersion-objective (WIO). This allows monitoring the chemical reaction progress in the water droplet by tracking the laser wavelength. A proof-of-concept demonstration of chemical sensor is performed by observing the process of salt dissolution in water and diffusion of two droplets with different RI. This WIO pumped sensing configuration provides a path towards an on-chip chemical sensor for studying chemical reaction kinetics in real time.

## 1. Introduction

Light whispering-gallery-modes (WGMs) in microresonators, which are the analogy of acoustic waves travelling in a closed concave surface such as St. Paul’s Cathedral, support ultrasmall mode volumes and ultrahigh quality (Q) factors. WGM microresonators with high local field intensities make them an excellent platform for enhancing the interaction between light and matter, based on which many applications have been demonstrated over the past two decades, such as low-threshold lasers [1,2], opto-mechanics [3,4], integrated optical devices [5,6], non-linear optics processes [7,8,9], and optical sensors [10,11,12]. Particularly, the WGM microresonator with strong evanescent waves has been proven as a versatile label-free biochemical sensor [13,14,15,16,17] with high sensitivity and low detection limit down to single molecules and nanoparticles by monitoring the shift [18], split [16,19,20,21], or broadening [22] of the resonance spectra. Various types of WGM microresonators including microsphere [18], microtoroid [16], microbubble [23,24], microdisk/ring [25], and microcapillaries [26,27] have been used in biosensing experiments; they can be catalogued into passive resonators and active resonators. In passive resonator-based sensor configuration, the resonator is driven by a waveguide, such as a relatively fragile tapered fiber or prism. The relative position between them must be adjusted precisely by a nano-positioning platform that makes the sensing system costly and cumbersome. While in active resonator sensor configuration, the fluorescence or laser spectrum is collected by free-space optics that makes the sensing system relatively practical. 

In most WGM microresonator (both for passive and active) based biochemical sensor configurations, the microcavity is immersed in water and sealed in a microfluidic cell. This sensing configuration is a closed system. To change the refractive index (RI) of liquid around the microresonator, a micro syringe pump is used to pump the biochemical sample under test through the microfluidic channel. Thus, this sensing configuration cannot change the RI of liquid around the microresonator and sensing it immediately because sensing samples must be crossed through the microfluidic channel. Here, we report on the demonstration of a biochemical sensor based on a water-immersion-objective (WIO) pumped on-chip microdisk laser. This WIO pumped configuration is an open sensing system. The microdisk is directly immersed in the water environment that is what the WIO requires. By monitoring the laser wavelength, any feeble disturbance of the RI of the water can be detected in real time. Thus, the WIO pumped configuration provides an excellent platform for monitoring the RI change of the water that is induced by chemical reactions. In this paper, we demonstrate that the salt dissolution in water process and diffusion process of two droplets with different RI can be tracked by the lasing mode wavelength in real time. We believe that this work opens up a novel on-chip biochemical sensor based on a WIO pumped configuration, which provides potential accesses to study chemical reaction kinetics.

## 2. Laser Operation 

### 2.1. Optical System Setup

**Figure 1:**
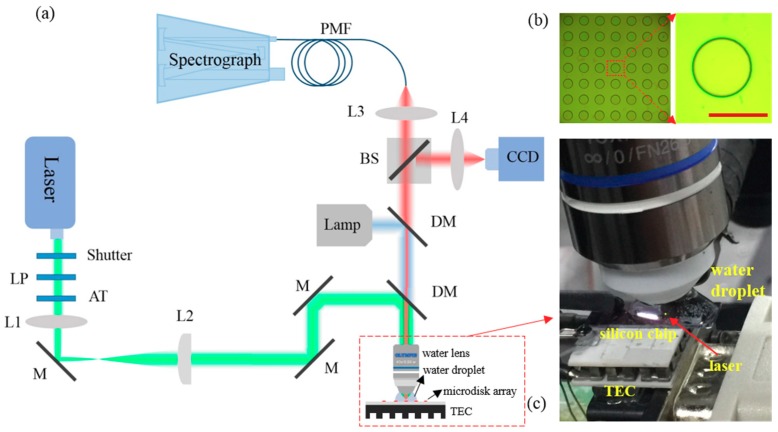
Optical system setup. (**a**) Schematic of lasing chemical sensor pumped by water-immersion-objective (WIO). LP: linear polarizer; AT: attenuator; L: lens; M: mirror; DM: dichroic mirror; BS: beam splitter; PMF: polarization-maintaining fiber. (**b**) Optical images of microdisk array and single microdisk, the scale bar is 40 μm. (**c**) Close-up image of the microdisk laser-based chemical sensor under WIO. The silicon chip is mounted on a home-made TEC which is connected to a temperature controller (Thorlabs, TED4015).

The optical system setup is shown in Figure 1a, which is a typical optically pumped microcavity laser setup, except that a water-immersion objective (WIO) instead of conventional air objective is used. The pump laser is focused on the active microdisk resonator, which is directly immersed in water, through this WIO (LUMPlanFLN 40× W, Olympus, Tokyo, Japan). In this study, an active SU-8 microdisk resonator doped with Rhodamine B (RhB) is used for the experiment. The microdisk laser array (Figure 1b) is fabricated on silica-on-silicon chip and the detailed fabrication process can be found in previous studies [28,29]. The diameter and thickness of the microdisk are controlled to 40 μm and 1 μm, respectively. To avoid thermal wavelength drift induced by the fluctuation of the lab temperature, the sample chip is mounted on a home-made TEC (TZET Co. Ltd., Tianjin, China). The TEC is connected to a temperature controller (TED4015, Thorlabs Inc., Newton, NJ, USA) with a controlling precision of 2 mK. The pump light used here is an ns-pulsed OPO laser with a repetition rate of 10 Hz. The wavelength of pump laser is tuned to 532 nm for matching the absorption band of RhB. The laser emitted from the active microdisk is collected through free-space and recorded by the monochromator (HORIBA iHR550, Kyoto, Japan) equipped with a cooled CCD detector. A 1200 mm^−1^ grating is installed in the monochromator.

### 2.2. Threshold Measurement and Spectral Identification

**Figure 2:**
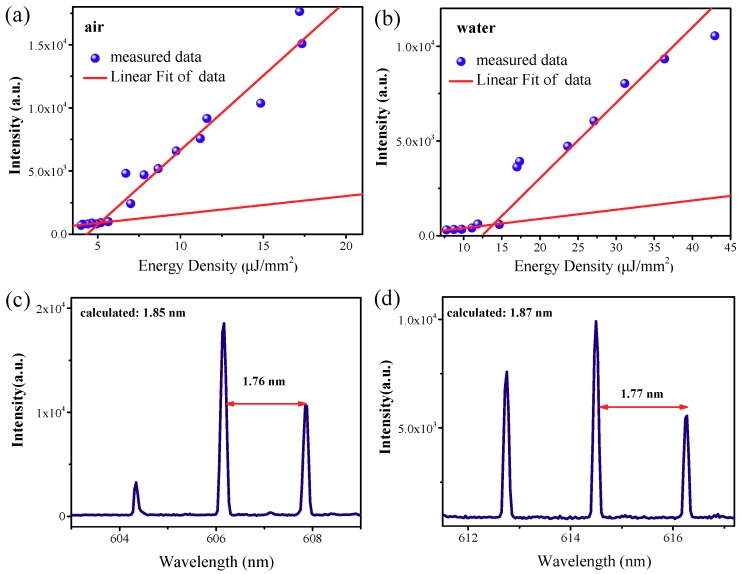
Comparisons of the output laser intensity as a function of the energy density (light-light curve) around the single mode lasing wavelength pumped by (**a**) air objective and (**b**) WIO, respectively. Typical laser spectrum pumped by air objective (**c**) and WIO (**d**), respectively. Calculated free spectral range (FSR) and measured FSR are both denoted.

The threshold of the laser emission is firstly measured by tuning the power of pump light and the light emission form the active microdisk is recorded correspondingly. Both the air objective (LMPlanFLN 20×, Olympus, Tokyo, Japan) pumped configuration and WIO pumped configuration are performed for comparison; the result is shown in Figure 2. Under both configurations, there are obvious kinks (Figure 2a,b), which indicate that the lasing thresholds are about 5.2 μJ/mm^2^ and 13.9 μJ/mm^2^, respectively. The linewidth of the laser peak above the threshold is about 0.08 nm. The corresponding Q factor is about 7800. However, this is not the real Q factor which is limited by the grating of the monochromator. The laser threshold pumped by WIO is higher than that pumped by the air objective. This is attributed to that the RI contrast is lower, which increases radiation loss in the WIO pumped configuration. 

To verify this, the eigenmodes of a microdisk surrounded by air and water are calculated by Finite Element Method (FEM) with COMSOL Multiphysics 3.5a [30]. The perfectly matched layers (PMLs) are introduced to accurately calculate the radiation-loss-related Q_rad_. The Q_rad_ of the fundamental radial mode (radial quantum number *p* =1) is 1.5×10^8^ when the microdisk is exposed in air, while it drops to 2.4×10^7^ when the microdisk is immersed in water.

**Figure 3:**
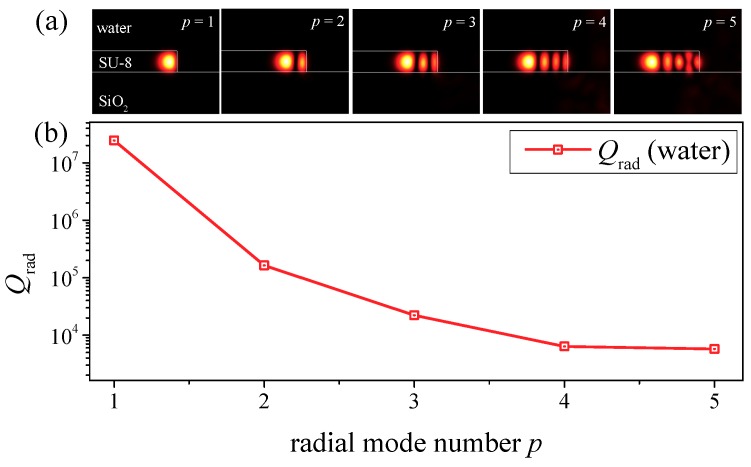
(**a**) The field distributions for different radial mode numbers. (**b**) Calculated radiation-loss-related Q_rad_ for different radial mode numbers.

The Q_rad_ and field distribution of different radial modes are calculated and plotted in Figure 3. The Q_rad_ decreases exponentially with the increment of *p*. Thus, we can conclude that the laser peaks shown in Figure 2c,d are the fundamental modes with adjacent azimuthal mode numbers. The free spectral range (FSR) is slightly larger in the WIO pumped configuration, because of the RI increment of surrounding materials of the microdisk. Little discrepancy between the calculated FSR and measured FSR may result from the inaccuracy of the microdisk diameter during the fabrication process.

## 3. Basic Element Sensing

### 3.1. Bulk Refractive Index Sensitivity (BRIS)

**Figure 4:**
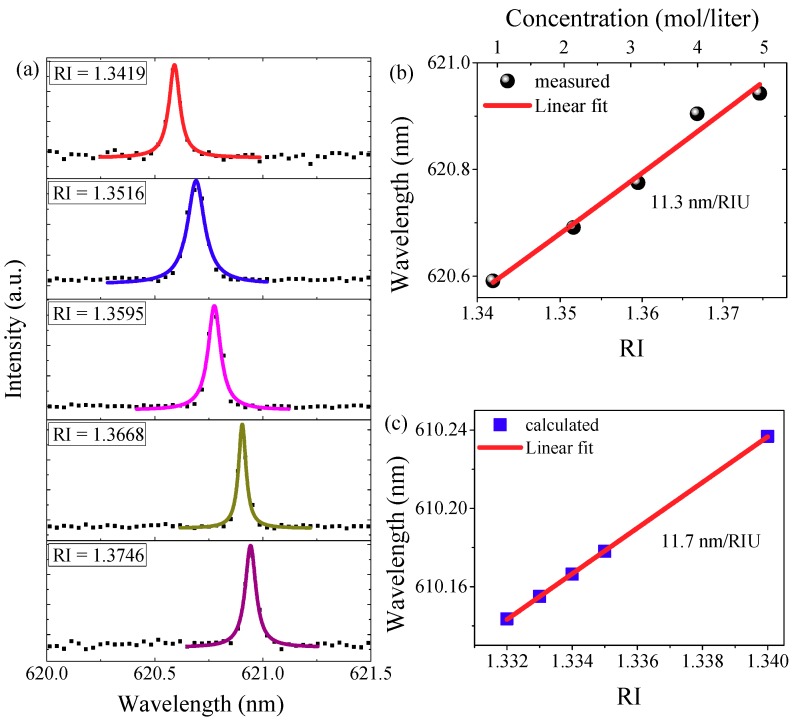
(**a**) Lasing spectrum shift by slightly increasing the refractive index (RI) of the water droplet. Insets show the RI of the NaCl solutions. (**b**) Measured wavelengths of the lasing modes as a function of the NaCl concentration (top *x*-axis) and RI of the water droplet (bottom *x*-axis); the measured data is linear fitted. (**c**) The calculated RI sensitivity.

Under the WIO pumped configuration, the microdisk is directly immersed in a water environment, so the lasing spectra will be quite sensitive to the RI of the water. The BRIS of the microdisk laser is tested by directly changing the RI of the water droplet between the WIO and the silicon chip. Different RI solutions are obtained by dissolving different masses of NaCl into deionized water. Five NaCl solutions with concentrations of 1, 2.05, 3, 4.03, and 4.94 mol/liter are prepared for testing as shown in Figure 4a. To change in advance each droplet between the WIO and silicon chip, the sample is washed by the deionized water at least twice to eliminate the error of the droplet RI. 

The lasing spectra are recorded for each droplet, respectively, which are shown in Figure 4a. The central lasing wavelength is obtained by fitting the lasing peaks with Lorentz function. The lasing wavelength shift is plotted as a function of RI (Figure 4b). By linear fitting the measured wavelength data, the slope, i.e., the BRIS is 11.3 nm/RIU. 

The wavelengths of the eigenmodes are calculated by changing the RI of the surrounding environment of the microdisk in the simulation model, and are also plotted over RI in Figure 4c. As shown, the calculated BRIS is 11.7 nm/RIU. The measured BRIS matches very well with this calculated value. The relatively high BRIS guarantees the performance of microdisk laser as a chemical sensor.

### 3.2. Thermal Sensing 

SU-8 material exhibits high thermal optics (TO) coefficient of −3.5 × 10^−4^ K^−1^ [31], which is higher than that of materials that are commonly used to fabricate WGM resonators, such as silica (1 × 10^−5^ K^−1^) [32] and silicon (1.8 × 10^−4^ K^−1^) [33]. Combining with on-chip integration, ease of laser probing and readout based on free-space optics against passive WGM thermal sensors using relatively fragile tapered microfiber [34,35], the fabricated all-polymer microdisk laser provides a good platform for ultrasensitive thermal sensing. Thus, the thermal sensing of the SU-8 microdisk laser is performed. 

**Figure 5:**
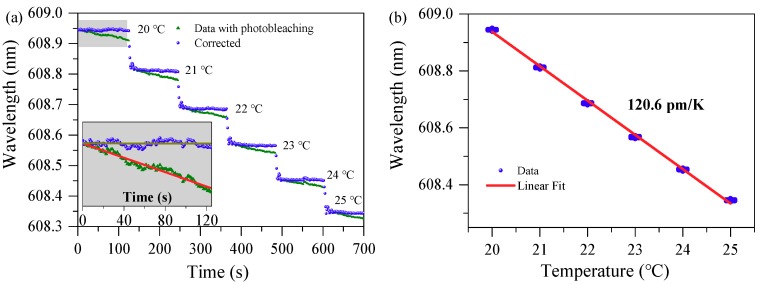
Temperature dependence of wavelengths of lasing mode. (**a**) Lasing emission wavelength shift with photobleaching and corrected data for photobleaching when changing the TEC temperature at a periodic interval. Inset shows the enlarged view at 20 °C. Brown and red line are the linear fitting curve. (**b**) Tracked laser wavelength with error bar vs temperature.

The laser emission spectra are recorded in real time when changing the TEC temperature by a step of 1 K. At each temperature, the spectra are recorded for about 2 mins. A home-made code is written to track the spectral positions of the laser peaks over time. The result of spectral shift over time with different temperatures is shown in Figure 5a, where the spectral shift (triangle dots) exhibits step change to the temperature. However, at each constant temperature, the lasing wavelength minor shifts linearly which can be seen from the enlarged view of spectral shift at 20 °C (inset in Figure 5a). This blue shift of laser wavelength is attributed to the decreased RI of the polymer-dye composites due to photobleaching [36,37,38,39]. As the spectral shift is linear over time [38], the spectral shift can be corrected by eliminating its slope (red fitting line in the inset of Figure 5a). The corrected spectral shift (circular dots) is almost flat over time at each constant temperature. 

The average values of laser wavelength with error bar for each temperature is plotted in Figure 5b. The linear fit result indicates the thermal sensitivity is as high as 120.6 pm/K and has benefited from the high TO coefficient. Here the thermal expansion effect is ignored since its coefficient of ~ 10^−6^ K^−1^ is much lower than TO coefficient [40]. 

For bio-chemical sensing, the temperature fluctuation and photobleaching will cause the resonance wavelength drift and influence the quantification of the proposed analytes. To eliminate the effect of temperature fluctuation, the sensor chip is mounted on a TEC for stabilizing the temperature. The wavelength drift caused by the photobleaching can be corrected by eliminating its slope. Recently, S. F. Wondimu also reported a novel scheme that allows for simultaneous compensation of temperature drift and photobleaching by using microdisk laser arrays with on-chip references [39]. 

## 4. Chemical Sensor

As the microdisk is directly immersed in the water droplet and the microdisk sensor exhibits high sensitivities, so the lasing mode can quickly sense the local change (e.g., RI) of the water droplet in real time. This provides an excellent platform for studying some chemical reactions in water, e.g., dissolution and diffusion process, by monitoring the laser wavelength. Thus in the following study, a proof-of-principle chemical sensor is demonstrated based on a WIO pumped configuration by adding salt crystals or NaCl solution into the water droplet. 

### 4.1. Salt Dissolution Process in Water

In this section, adding a certain quality of salt crystal into the water droplet between the WIO and silicon chip is performed. The water droplet with a fixed volume of 0.2 mL is firstly injected into the space between the WIO and silicon chip by a pipette. By tuning one microdisk just under the focus of pump light, the laser is generated above the threshold and the emission spectrum is automatically recorded with integration time of 1 s. Then salt crystal with a mass of 14 mg is cast into the edge of the droplet with a tweezer. In this process, the acquisition of emission spectra is ongoing for about 7 mins. 

**Figure 6:**
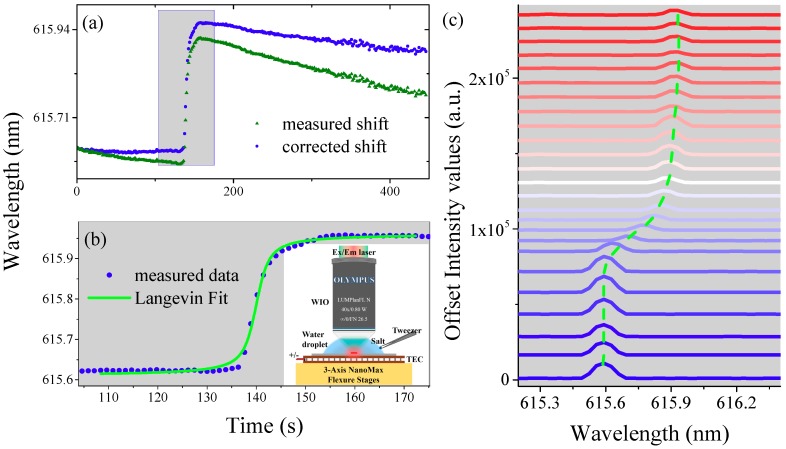
Monitoring the process of dissolution of salt in water using the lasing chemical sensor. (**a**) Measured lasing wavelength and corrected lasing wavelength shifts when adding the 14 mg salt in water under WIO. (**b**) Close-up view of corrected lasing wavelength and Langevin fitting. Inset shows the schematic diagram of operation process. (**c**) Dynamic change of lasing spectra in the process of dissolution of salt in water. The blue line (initial position) to red line (end position) is guided by the green dotted line.

After data acquisition, one laser peak is monitored and its spectral wavelength shift is shown in Figure 6a (triangle dots). Slow linear change of wavelength induced by photobleaching is observed, after correction, and the spectral shift is denoted by circular dots. Before casting salt into the droplet, the lasing wavelength is quiet, which can be seen from Figure 6a (circular dots). After casting salt into the droplet, the lasing wavelength is red shifted dramatically. Then the lasing wavelength blue shifts slowly and finally approaches to equilibrium over 400 s. The whole process is explained below. 

Once the salt is cast into the droplet, the salt will dissolve and diffuse in the water, which induces the increment of RI of the droplet. As a result, the spectrum shifts to longer wavelength dramatically. Since the salt crystal is cast in one side of the droplet, there exists a gradient in concentration of NaCl solution from this side to the other side. Therefore, the RI in the surrounding volume of the lasing microdisk first increases and then decreases with the progress of diffusion. Consequently, the spectrum starts to shift slowly to shorter wavelength and finally no longer changes at the end of diffusion process (> 400 s), as shown in Figure 6a. 

A close-up view of the dramatic change of laser wavelength is shown in Figure 6b. The trajectory of spectral shift is fitted well by Langevin function, which is written as: L(t) = coth(t) − 1/t, where the "coth" is the hyperbolic cotangent, defined as coth(t) = (e^t^ + e^−t^)/(e^t^ − e^−t^). The dynamic change of the laser spectra is also plotted in Figure 6c (guided by the dotted line). 

**Figure 7:**
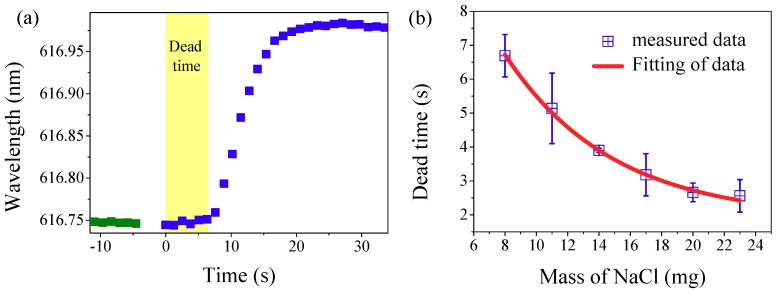
(**a**) Response of lasing wavelength recorded separately before (green dots) and after (blue dots) adding the salt (8 mg) into the water. The yellow region represents the dissolution process (dead time). (**b**) Dead time as function of the mass of the salt.

We found that the spectral does not shift as soon as the salt is cast into the droplet. From adding the salt into the droplet, the laser wavelength is almost stable for a certain time until the wavelength shifts obviously. Here, this time is named as dead time. In order to measure the dead time, the spectral data are recorded separately before and after adding the salt (8 mg) into the droplet, which is shown in Figure 7a. The time to add the salt into the droplet is set to 0 s. The dead time is about 6.5 s, as shown in Figure 7a (denoted as a yellow region). After the dead time, the laser wavelength drifts steeply. Dead time as a function of salt mass is also investigated and plotted in Figure 7b. As shown, the dead time decrease exponentially with the salt mass. This is attributed to the heavier salt possessing a larger surface area and the dissolution process is shorter. 

Adding salt into the water actually contains two processes: dissolution of salt and diffusion of NaCl solution. The onset of the spectral shift means the RI of the surrounding volume of the lasing microdisk starts to change due to the diffusion of NaCl solution. The dead time is the sum of salt dissolution time and diffusion time of NaCl solution from one side of the droplet to the middle (where the lasing microdisk locates) of the droplet. Therefore, we perform next experiment by injecting NaCl solution directly into the water droplet to investigate the diffusion time.

### 4.2. Diffusion Process of Two Different Solutions

In this section, the diffusion process of two different RI solutions is investigated. The water droplet with a fixed volume of 0.1 mL is firstly injected to the volume between the WIO and silicon chip. Then 0.1 mL NaCl solution with RI of 1.3516 is quickly injected into the water droplet by a pipette tip. 

**Figure 8:**
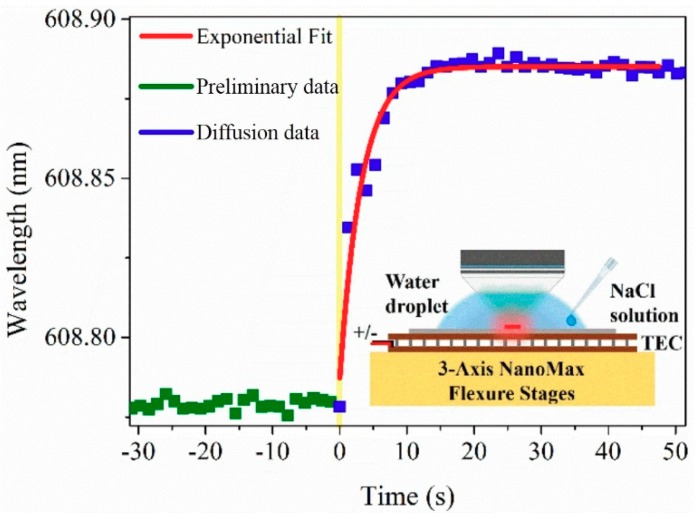
Monitoring the process of mixing NaCl solution with water using the lasing chemical sensor. The yellow line represents the diffusion process. Inset shows the schematic diagram of operation process.

One laser peak was monitored and its spectral shift is shown in Figure 8. The spectral data are recorded separately before and after injecting NaCl solution into the droplet. The spectral response is different from that in adding salt into the droplet. As soon as the NaCl solution is injected into the droplet, the laser wavelength shifts immediately. This indicates the RI of the surrounding volume of the lasing microdisk changes immediately due to the diffusion of NaCl solution, and this diffusion process is very fast (less than 1 s as shown in Figure 8). Therefore, it can be confirmed that the dead time in Figure 7a is the reaction time of salt dissolution in water. 

## 5. Conclusion

We introduced and systematically studied on-chip versatile chemical sensors based on a WIO pumped WGM microlaser in which the microdisk is directly immersed in the water droplet. Laser properties including threshold and spectrum are firstly investigated and also compared with the microdisk pumped by WIO and air objective, respectively. Lasing mode identification is also performed by using FEM. Then, basic physical elements of RI and temperature sensing are tested, which shows that BRIS is as high as 11.3 nm/RIU and the thermal sensitivity can reach 120.6 pm/K. Finally, a proof-of-concept demonstration of the chemical sensor is carried out by locally changing the droplet RI with the help of typical chemical reactions. The tracking of the progression of dissolution and the diffusion process in real time are realized by monitoring the laser spectral shift. 

We anticipate that our demonstration can be further extended to study various biochemical reactions, such as endothermic and exothermic reactions. The application of the WIO pumped configuration can be also broadened to multifunctional biological monitoring and treatment by directly immersing cells or tissues into the WIO droplet.
